# Intermittent and periodic fasting: molecular mechanisms, health benefits and research challenges

**DOI:** 10.3389/fnut.2026.1915344

**Published:** 2026-09-15

**Authors:** Ya Liu, Hongwei Guo

**Affiliations:** 1 School of Graduate Studies, Heilongjiang University of Chinese Medicine, Harbin, Heilongjiang, China; 2 College of Basic Medical and Sciences, Heilongjiang University of Chinese Medicine, Harbin, Heilongjiang, China

**Keywords:** cellular repair, clinical translation, healthspan, intermittent fasting, metabolic reprogramming, periodic fasting

## Abstract

Intermittent fasting (IF) and Periodic fasting (PF) are biologically plausible non-pharmacological interventions. They trigger core metabolic reprogramming from glucose catabolism to ketogenesis and activate conserved nutrient-sensing networks including AMPK and sirtuins, coupled with mTOR/IGF-1 axis suppression. Such adaptive regulation enhances cellular repair, antioxidant defense and inflammatory homeostasis, conferring multi-system benefits encompassing metabolic improvement, neuroprotection, anti-tumor activity and healthspan extension. Clinically, time-restricted eating (TRE) and fasting-mimicking diets (FMDs) have demonstrated feasibility and short-term efficacy in pilot clinical studies, although their long-term translational potential and durability of benefits remain unproven. However, current research faces major bottlenecks: over-reliance on homogeneous animal models, insufficient high-quality long-term human trials, unresolved individual response heterogeneity, and the lack of validated non-invasive biomarkers and systematic long-term safety evidence. This mini-review systematically elaborates the core regulatory mechanisms of IF and PF through a hierarchical framework that distinguishes primary drivers from secondary consequences and speculative mechanisms, critically evaluates the evidence base across model systems, analyzes key translational barriers, and proposes a prioritized research agenda. Our objective is to provide a conceptually grounded synthesis that identifies genuine scientific uncertainty, distinguishes established knowledge from speculation, and offers a perspective capable of guiding future investigation toward precise clinical translation.

## 1 Introduction

IF and PF have become among the most actively investigated non-pharmacological strategies for healthspan extension and age-related disease prevention over the past two decades. Defined by structured feeding-fasting cycles rather than continuous energy reduction, intermittent fasting typically involves repetitive fasting intervals of 12–48 h, whereas PF implements longer fasting episodes exceeding 48 h at low monthly frequencies (1, 2). Compared with chronic caloric restriction, these timing-dependent regimens may offer improved population adherence in some settings, potentially making them more feasible for long-term implementation (3, 4). The distinction from caloric restriction is conceptually important because intermittent and PF regimens emphasize meal timing and fasting duration rather than total energy intake.

The past decade has witnessed exponential growth in fasting-related publications, spanning molecular mechanisms in model organisms, observational studies, and controlled clinical trials. This research has established that fasting triggers a metabolic transition from glucose-dependent catabolism toward fatty acid oxidation and ketogenesis, activating nutrient-sensing pathways including AMPK, sirtuins, and mTOR/IGF-1 axis modulation (5, 6). Benefits have been reported across multiple systems: improved metabolic health, neuroprotection involving increased BDNF expression, enhanced cellular repair through autophagy, anti-tumor activity, and potential lifespan extension (7–12).

In contrast to previous descriptive reviews that primarily catalog molecular pathways without critical weighting, our contribution provides three distinctive advances: (1) a hierarchical framework that distinguishes primary drivers from secondary consequences and speculative mechanisms, enabling identification of the most therapeutically relevant targets; (2) explicit evidence-grading for each mechanism across model systems, which exposes substantial gaps between preclinical findings and human validation; and (3) systematic identification of translational barriers with a prioritized research agenda. This conceptual structure moves beyond the enumerative approach that characterizes the majority of existing summaries (13–15).

However, the field currently lacks a critical synthesis addressing three pressing questions essential for advancing from descriptive characterization to evidence-based clinical application. First, which mechanisms represent primary drivers of fasting-induced adaptations versus secondary responses? Second, to what extent do preclinical findings translate to human physiology? Third, what evidence gaps most critically impede clinical translation? This review addresses these questions by providing a conceptually structured synthesis that moves beyond existing descriptive accounts. We propose a hierarchical framework distinguishing primary adaptive responses from downstream consequences, critically evaluate evidence strength across model systems, analyze persistent methodological limitations, and articulate a prioritized agenda for future research (Figure 1).

**Figure 1:**
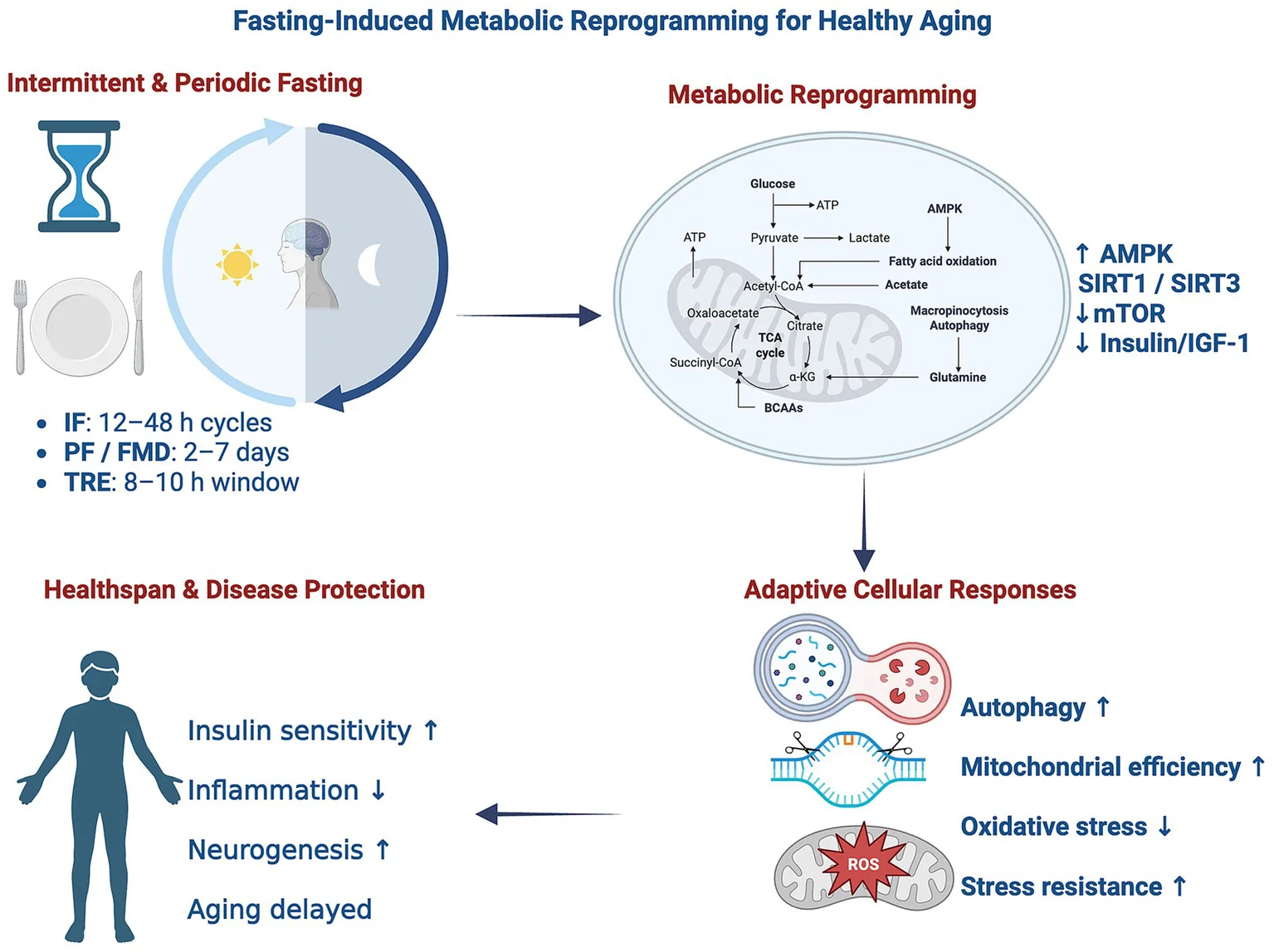
Proposed conceptual framework of fasting-induced metabolic reprogramming for healthy aging. This figure summarizes the current understanding of how IF, PF, and time-restricted eating (TRE) may influence metabolic and cellular pathways associated with healthy aging. Reduced nutrient availability is proposed to promote metabolic reprogramming through activation of AMPK and sirtuins and suppression of insulin/IGF-1 and mTOR signaling, which are associated with enhanced fatty acid utilization, autophagy, mitochondrial function, oxidative stress regulation, and cellular resilience. These adaptive responses are thought to contribute to improvements in insulin sensitivity, inflammation, neuroprotection, and healthy aging. The diagram represents a conceptual synthesis of current evidence and is intended as a heuristic framework rather than a validated linear causal pathway. The timing, magnitude, and interactions of these responses are context-dependent and may vary across tissues, fasting regimens, and species.

## 2 A hierarchical framework for fasting-induced adaptive mechanisms

The biological effects of intermittent and PF emerge from an integrated network of molecular, cellular, and systemic adaptations. However, the literature often presents these mechanisms as equivalently important, obscuring hierarchical relationships. We propose a framework distinguishing three levels of fasting-induced mechanisms organized by temporal sequence and causal relationship to nutrient deprivation. Evidence categories used throughout this review: “Well-established” mechanisms have consistent evidence across multiple species including human studies with validation through genetic or pharmacological interventions; “Probable” mechanisms have strong preclinical support but limited human evidence; “Established” systemic effects have consistent clinical trial data; “Correlative” effects are consistently observed but causation is unproven; “Speculative” mechanisms have preliminary or no direct evidence and require substantial investigation.

Importantly, the hierarchical relationships are not static but exhibit dynamic crosstalk varying across fasting duration, protocol type, tissue context, and species. These dynamic interactions, rarely dissected in the literature, are critical for understanding how fasting regimens of different durations and frequencies produce distinct physiological outcomes (Figure 2).

**Figure 2:**
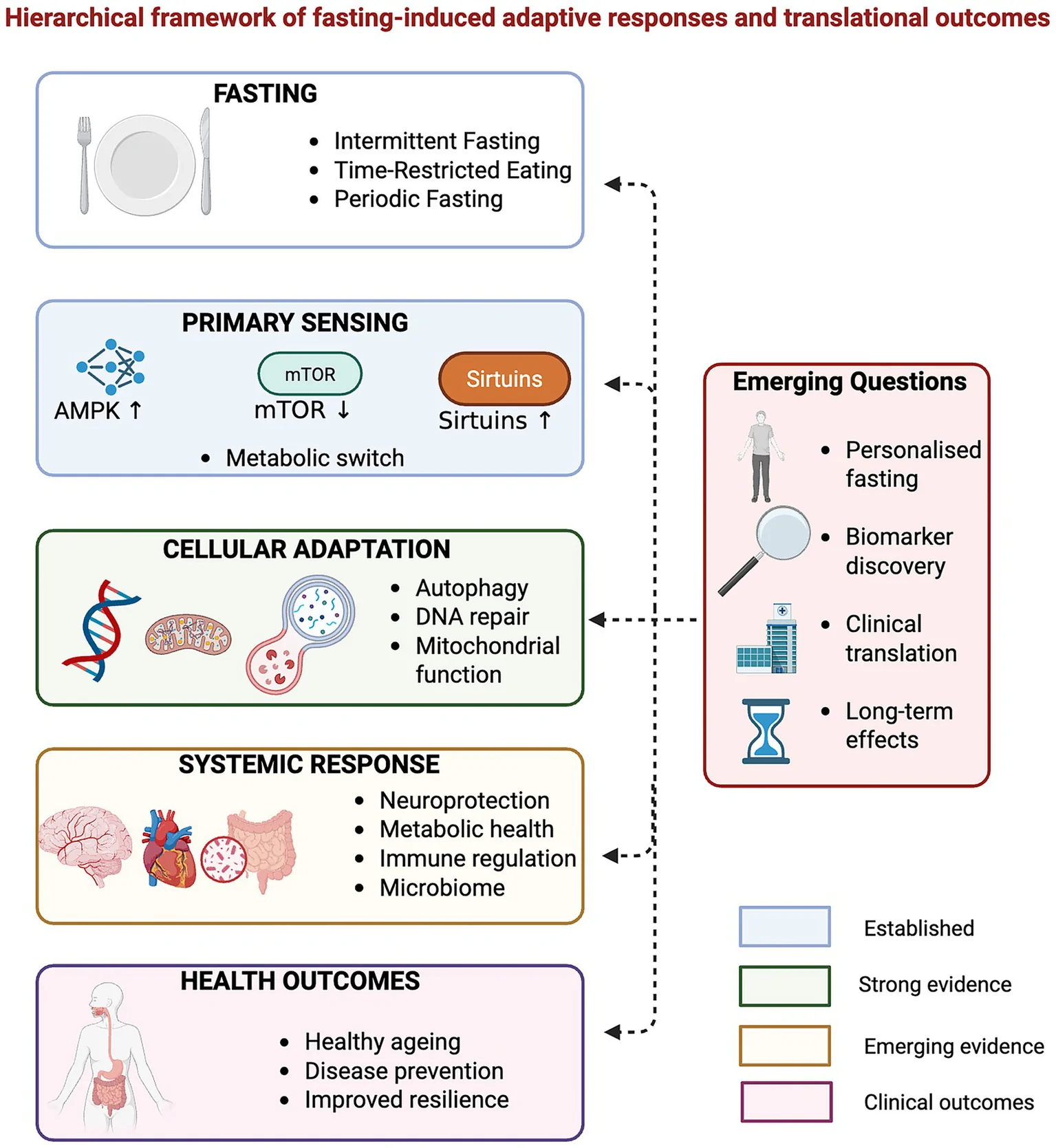
Proposed hierarchical framework of fasting-induced adaptive responses and translational outcomes. This figure presents a hierarchical conceptual framework summarizing the current understanding of fasting-induced biological adaptations and their potential physiological and translational outcomes. Fasting is associated with activation of nutrient-sensing pathways (AMPK, mTOR, and sirtuins), followed by adaptive cellular processes including autophagy, DNA repair, mitochondrial remodeling, and oxidative stress regulation. These responses are linked to systemic adaptations involving metabolic regulation, immune function, neuroprotection, and microbiome modulation, ultimately contributing to improved physiological resilience, healthy aging, and disease prevention. The side panel highlights key translational priorities and unresolved questions. The framework synthesizes current evidence into a hierarchical representation of these adaptive responses, recognizing that the interactions depicted are dynamic, context-dependent, and may vary across tissues, fasting regimens, species, and the available evidence.

### 2.1 Primary adaptive responses

The most established primary response to fasting is the metabolic switch from glucose to ketone utilization. Following 12–16 h of fasting in humans, hepatic glycogen reserves become depleted, driving a fundamental metabolic transition away from glycolysis toward enhanced fatty acid oxidation and ketogenesis (16–18). This adaptive shift elevates circulating ketone bodies, including β-hydroxybutyrate and acetoacetate, which serve as alternative energy substrates. Beyond serving as metabolic fuel, β-hydroxybutyrate functions as a critical signaling mediator with histone deacetylase inhibitory properties, activating Nrf2-dependent antioxidant signaling, suppressing NLRP3 inflammasome activation, elevating BDNF expression, and strengthening cellular stress tolerance (19, 20).

Concurrently, reduced circulating glucose and insulin concentrations attenuate insulin and IGF-1 signaling, alleviating metabolic overload in insulin-sensitive organs and suppressing anabolic proliferative pathways (21). This attenuation constitutes an evolutionarily conserved pro-longevity axis linked to improved metabolic homeostasis, reduced malignant susceptibility, and enhanced stress resilience (22, 23).

Nutrient deficiency reduces intracellular ATP and elevates AMP, leading to robust AMPK activation. Acting through both allosteric activation and phosphorylation by upstream kinases, AMPK accelerates catabolic processes while restraining anabolic pathways through mTORC1 suppression (24). Fasting also increases the NAD^+^/NADH ratio, activating NAD^+^-dependent deacetylases including SIRT1 and SIRT3. SIRT1 promotes mitochondrial biogenesis, stress resistance, autophagic clearance, and genomic stability via deacetylation of PGC-1α, FOXO transcription factors, and p53. SIRT3 optimizes mitochondrial respiratory function and antioxidant defense (25). Although AMPK activation is consistent in rodents, several human trials report no significant change in muscle AMPK phosphorylation after 12-h fasting, and genetic variability in the PRKAA1 locus modulates the magnitude of response. Such null results are rarely foregrounded but indicate that AMPK is not universally required for fasting effects in all contexts.

Reduced amino acid availability and diminished growth factor signaling synergistically inhibit mTORC1, representing a central driver of fasting-mediated protection by promoting autophagy, restraining protein synthesis, and enhancing stress adaptation (26, 27). In *Caenorhabditis elegans*, RHEB-1-dependent signaling mediates IF-associated longevity extension (28).

*Evidence strength assessment*: The metabolic switch and AMPK/sirtuin activation constitute well-established mechanisms supported by consistent evidence across multiple model systems and species. Suppression of insulin and IGF-1 signaling is similarly robust. These mechanisms have been validated through pharmacological manipulation, genetic interventions, and detailed time-course studies. Translation from animal models to humans is supported by consistent observations in human fasting studies. However, the extent of pathway crosstalk and relative contribution of each pathway to specific outcomes remain incompletely characterized.

#### 2.1.1 Pathway crosstalk: dynamic interactions across fasting durations and species

The interactions between primary fasting responses are dynamic and context-dependent. We propose a causal hierarchy in which the metabolic switch (ketogenesis) and AMPK activation serve as primary drivers with the largest direct causal contribution; mTOR/IGF-1 suppression and sirtuin activation exert moderate primary effects; while secondary processes such as autophagy and mitochondrial remodeling are consequential amplifiers rather than independent initiators.

Kinetic and comparative data illustrate this hierarchy. In rodents, AMPK phosphorylation peaks at 4–6 h of fasting, preceding measurable mTOR suppression (observed at 8–10 h). In humans, these responses are more temporally coupled, with both occurring within 12–16 h. In liver tissue, SIRT1 activation is heavily dependent on AMPK-mediated NAD^+^ availability; in neural tissue, however, sirtuin activation appears partially AMPK-independent, suggesting tissue-specific regulatory logic. Across species, the AMPK-mTOR-sirtuin axis exhibits different magnitudes: in mice, hepatic AMPK activity increases 3- to 5-fold after 24 h, whereas in human muscle biopsies the increase is more modest (approximately 1.5- to 2-fold). These kinetic and tissue-specific differences underscore that simple linear statements (“fasting activates X → suppresses Y → produces benefit”) are inadequate and may misrepresent the integrated physiology.

### 2.2 Secondary cellular processes

Autophagy induction represents one of the most consequential secondary responses. Suppressed mTORC1 activity, AMPK activation, and reduced amino acid availability converge to stimulate autophagic clearance of damaged organelles and proteins (20, 29). This process is essential for maintaining cellular homeostasis, particularly in post-mitotic tissues. Autophagy deficiency abolishes lifespan-extending effects of IF in Drosophila and *C. elegans* (30, 31). In humans, fasting upregulates autophagy-related genes including LAMP2, LC3B, and ATG5 (32, 33). Circadian rhythmicity of autophagy is notable, with time-restricted feeding leveraging this natural oscillation.

However, evidence for autophagy induction in humans is substantially weaker than in animal models. Direct measurement of autophagic flux *in vivo* remains challenging; most human studies rely on surrogate markers such as LC3B-II/LC3B-I ratios or p62 levels in peripheral blood mononuclear cells. These markers indicate changes in autophagy-related protein expression but do not reliably measure flux. A persistent debate surrounds whether observed increases reflect enhanced flux or impaired clearance. Limited tissue accessibility compounds this uncertainty, as circulating markers may not reflect autophagic activity in key tissues.

Mitochondrial adaptations constitute another critical secondary response. Coordinated activation of AMPK, SIRT1, and PGC-1α enhances mitochondrial biogenesis and oxidative metabolic efficiency while reducing ROS generation (34). This remodeling improves metabolic flexibility (35). Fasting reinforces genomic stability by enhancing DNA repair mechanisms through AMPK, SIRT1, and mTORC1 inhibition, reflected by diminished oxidative DNA damage (36, 37). Extended fasting regimens facilitate stem cell-dependent tissue regeneration within immune and intestinal compartments by suppressing PKA signaling and cyclin D1 expression (38). Elevated spermidine abundance during fasting establishes a favorable microenvironment for regenerative restoration (39).

Fasting reinforces endogenous antioxidant defense systems by upregulating enzymatic effectors including SOD, catalase, and glutathione peroxidase through Nrf2-ARE axis activation. Ketone bodies directly potentiate Nrf2 activity (40). Enhanced mitochondrial efficiency limits ROS production, creating a positive feedback loop. In preclinical models, IF decreases circulating malondialdehyde while elevating glutathione and SOD (41, 42).

*Evidence strength assessment*: Autophagy induction and mitochondrial remodeling are probable mechanisms with strong preclinical support but limited direct evidence in humans. The magnitude, tissue-specificity, and functional significance of autophagy induction in humans remain poorly characterized. Mitochondrial adaptations are well-documented in animal studies and supported by indirect evidence in humans. The challenge of measuring these processes non-invasively represents a significant barrier to establishing their role in clinical benefits. The flux-versus-marker debate deserves explicit scrutiny. Increases in LC3B-II/LC3B-I ratios or decreases in p62 levels in peripheral blood mononuclear cells (PBMCs) are commonly interpreted as enhanced autophagy, but these changes can equally reflect impaired autophagosome clearance, transcriptional upregulation unrelated to flux, or post-translational modifications that do not correlate with degradative activity. Direct measurement of autophagic flux requires lysosomal inhibition or isotopic tracer methodologies that are invasive and rarely applied in humans. Tissue-level human measurements are virtually absent; no validated circulating biomarker of autophagic flux exists. The functional significance of observed marker changes for clinical outcomes; such as reduced inflammation or improved insulin sensitivity, therefore remains uncertain. The preclinical-to-human gap for autophagy is particularly wide, and studies that rely solely on PBMC markers should be interpreted with caution.

### 2.3 Tertiary systemic effects

At the organismal level, fasting exerts multi-system benefits through coordinated tertiary effects. Inflammatory resolution occurs through multiple complementary pathways. Fasting reduces proinflammatory mediators including TNF-α, IL-1β, and IL-6, while anti-inflammatory factors including IL-10 and adiponectin are augmented (43, 44). β-hydroxybutyrate-mediated NLRP3 inflammasome suppression, M2 macrophage polarization, and diminished neutrophil infiltration collectively restrain inflammatory progression (45). Fasting reshapes gut microbial composition toward an anti-inflammatory phenotype (46). However, the extent to which inflammatory changes are mediated by weight loss versus fasting-specific effects remains uncertain, and clinical significance has not been established.

Metabolic improvement constitutes another pivotal benefit. Insulin sensitivity is enhanced through reduced basal insulin secretion, accelerated peripheral glucose uptake, and suppressed hepatic gluconeogenesis (38, 43, 47). Fasting optimizes adiponectin-leptin balance (33, 48). Hepatic FGF21 expression is robustly induced, potentiating fatty acid oxidation, ketogenesis, and insulin responsiveness (14, 25). Although acute fasting stimulates GH secretion, prolonged fasting exerts favorable regulatory control over the GH/IGF-1 axis (48, 49). Weight loss is not universal; compensatory hyperphagia during refeeding occurs in a subset of individuals, and reductions in resting energy expenditure can partially offset caloric deficits. Some randomized trials show no significant cognitive improvement with IF, and others report increased cortisol or menstrual cycle disruption, particularly in premenopausal women.

Fasting exerts profound effects on the nervous system. IF increases BDNF availability to sustain neuronal survival, synaptic integrity, and cognitive performance (50–52). Fasting promotes neurogenesis and synaptic plasticity, strengthening resilience against neurodegenerative disorders through attenuated protein aggregation and enhanced autophagy (53, 54).

Both intermittent and PF remodel gut microbial community structure, increasing Bacteroidetes while reducing Firmicutes, accompanied by enrichment of beneficial genera including Lactobacillus, Akkermansia, and Christensenellaceae (46, 55). Remodeled microbiota generate protective metabolites including short-chain fatty acids that strengthen intestinal barrier integrity, suppress inflammation, and modulate systemic energy metabolism (39, 46).

*Evidence strength assessment*: These systemic effects are documented in both preclinical and clinical studies, though substantial inter-individual variability exists. Metabolic health benefits are supported by consistent findings from randomized controlled trials. Neuroprotective effects are strongly supported by animal studies and preliminary human evidence, though definitive clinical trials with cognitive outcomes are limited. Microbiome remodeling is consistently observed but the causal contribution of microbial changes to systemic benefits remains correlative. The magnitude of tertiary effects is influenced by baseline health status, genetic background, and environmental factors.

### 2.4 Speculative and emerging mechanisms requiring substantial investigation

Several proposed mechanisms remain hypothetical, with insufficient evidence for firm conclusions. We evaluate the quality of supporting data as follows:

Epigenetic memory: supported by only 2 rodent studies; persistence beyond the fasting period is unknown, tissue-specificity is uncharacterised, and human data are absent.

Xenohormesis (phytochemical amplification): zero direct experimental evidence; the hypothesis is biologically plausible but entirely untested (56).

Long-term circadian reprogramming: supported by animal clock-gene data, but human studies are limited to short-term meal-timing shifts; durability and health impact in free-living populations remain unclear (57).

Causal microbiome mediation: although compositional shifts are consistent, FMT experiments in mice are preliminary and have yielded inconsistent results; causation of systemic benefits in humans is unproven.

Mitohormesis and polyamine dynamics: both have preclinical support but lack dose–response characterisation and human validation (58, 59).

*Risks of overinterpretation*: Premature incorporation of these speculative mechanisms into review articles and grant proposals can create a false impression of established knowledge, potentially diverting research resources toward untested hypotheses. In popular discourse, such overinterpretation can lead to unwarranted confidence in unproven protocols and premature adoption by the public. We urge authors, clinicians and journalists to apply rigorous scrutiny and clearly distinguish these hypotheses from mechanistically validated pathways. Table 1 summarizes the evidence basis and key limitations of fasting-related mechanisms.

**Table 1:** Evidence strength summary of intermittent fasting/PF-induced adaptive mechanisms.

Mechanism	Evidence strength	Preclinical support	Human support	Key limitations
Metabolic switch (ketogenesis)	Well-established	Consistent across species	Consistent across studies	Temporal dynamics vary; individual variability
AMPK activation	Well-established	Multiple models, pharmacological validation	Supported by human studies	Tissue specificity unclear; magnitude varies
SIRT1/SIRT3 activation	Well-established	Strong mechanistic evidence	Supported by human studies	NAD^+^ availability complex; tissue specificity
mTOR/IGF-1 suppression	Well-established	Genetic and pharmacological evidence	Supported by human studies	Relative contribution uncertain
Autophagy induction	Probable	Strong genetic evidence in models	Limited; marker-based	Flux measurement challenging; marker vs. flux debate
Mitochondrial remodeling	Probable	Extensive animal data	Indirect evidence only	Human tissue access limited
Nrf2 antioxidant response	Probable	Strong animal evidence	Limited human data	Tissue-specific activation unclear
Inflammation resolution	Established	Extensive evidence	Consistent clinical data	Weight loss confounding; clinical significance
Metabolic improvement	Established	Extensive evidence	Strong RCT evidence	Durability uncertain; comparator issues
Neuroprotection	Probable	Strong animal evidence	Preliminary human data	Clinical outcomes lacking
Microbiome remodeling	Correlative	Consistent findings	Consistent findings	Causality unproven; mechanisms unknown
Epigenetic memory	Speculative	Preliminary data	Very limited	Persistence unknown; functional significance unclear
Xenohormesis	Highly speculative	No direct evidence	No evidence	Hypothesis untested
Circadian reprogramming	Speculative	Animal evidence	Very limited	Durability unknown in humans
Causal microbiome effects	Speculative	Preliminary transplantation data	Limited	Causality unproven; mechanisms unknown

## 3 Evaluating the evidence base: strengths, limitations, and translational gaps

### 3.1 Preclinical models: mechanistic power and translational limitations

Invertebrate models (*C. elegans* and Drosophila) have established fundamental mechanisms underlying fasting benefits. Studies in *C. elegans* have identified RHEB-1 signaling in IF-induced longevity, autophagy requirement for lifespan extension, and microRNA machinery regulation of fasting-responsive gene expression (28, 60). Drosophila studies established that circadian-regulated autophagy mediates intermittent time-restricted feeding benefits and identified TOR-independent pathways (61). The power of these models lies in genetic tractability and high-throughput screening. However, they provide limited insight into complex mammalian physiology. Metabolic differences and fasting tolerance limit direct applicability to human physiology. Lifespan extension in invertebrates does not necessarily predict effects in mammals.

Rodent studies represent the predominant preclinical evidence base. IF in rodents reduces adiposity, improves glucose homeostasis, delays tumorigenesis, and extends lifespan in some genetic backgrounds (62, 63). Studies have identified tissue-specific responses including hepatic ketogenesis, adipose lipolysis, muscle insulin sensitization, and neuronal stress resistance.

However, significant limitations constrain translational relevance. Most studies use young, male, genetically homogeneous inbred strains lacking genetic diversity, comorbidities, and environmental influences characteristic of human populations. Fasting protocols vary widely, obscuring dose–response relationships. Rodent metabolic rates substantially exceed humans, with corresponding differences in fasting physiology and tolerance. Many studies focus on acute effects while long-term adaptations remain poorly characterized. Temporal scaling is not straightforward: a 24-h fast in a mouse represents ~5–7% of its lifespan, whereas in a human it represents <0.01%.

Genetic variability in fasting responses represents a particularly important limitation. Studies in genetically diverse mouse populations reveal substantial differences in dietary restriction effects on cognition (64). Every-other-day feeding extends lifespan but fails to delay many aging symptoms (65). The underrepresentation of female rodents is another significant limitation, given that females show distinct metabolic and hormonal responses. Limited investigation of aging contexts fails to address how efficacy differs when interventions are initiated in middle or old age (66, 67).

### 3.2 Human studies: clinical relevance and methodological constraints

Human studies encompass observational research, controlled trials of TRE, and FMD trials. Observational studies of religious fasting have provided insights into feasibility and effects, though lack of controlled dietary intake limits causal inference. TRE has been the most extensively studied protocol; multiple RCTs have demonstrated efficacy for weight loss, glycemic control, and cardiovascular risk reduction, with effects comparable to or exceeding continuous caloric restriction (68, 69). FMDs have demonstrated feasibility and safety in multiple clinical contexts (23), reducing biomarkers of aging and improving metabolic health. Clinical trials in cancer patients demonstrate PF during chemotherapy is feasible and may enhance efficacy while reducing side effects (70).

However, human evidence faces substantial methodological constraints. Most trials are short-term, capturing acute adaptations but missing long-term outcomes. The near-impossibility of placebo-controlled designs complicates attribution of effects to fasting versus behavioral changes. Substantial individual variability in responses is not well characterized, and contributing factors require systematic investigation.

### 3.3 Inconsistent and negative findings

A critical limitation is the underreporting of inconsistent and negative findings. Genetic variability studies reveal substantial differences in fasting responses; some strains show negligible or damaging effects (64). Every-other-day feeding extends lifespan but fails to delay many aging symptoms (65). Growth hormone receptor-deficient mice fail to respond to IF (71). Sex differences are inadequately explored (66, 67). Age-dependent responses differ substantially; older animals may show blunted responses or adverse effects (37, 72). Human trials show substantial response variability; some individuals show minimal or no benefit. Some studies report compensatory hyperphagia undermining weight loss. Others document reductions in resting energy expenditure counteracting caloric restriction effects. Null or negative results; including no significant cognitive improvement in some trials, increased cortisol, menstrual cycle disruption, or reduced bone density, are often mentioned in passing but rarely foregrounded.

### 3.4 Critical knowledge gaps

Temporal and tissue-specific dynamics of key pathways are poorly mapped. Activation, interactions, and adaptation of pathways over time across tissues remain unclear. While gut microbiota shifts are consistently documented, causal role in mediating systemic benefits remains largely correlative. Epigenetic consequences of fasting lack characterization in terms of persistence, tissue-specificity, and long-term function. Circadian integration nuances require deeper exploration. Mechanistic impact of refeeding composition on sustaining benefits is understudied. Optimal hormetic “dose” remains undefined. Extensive crosstalk and redundancy obscure individual mechanism contributions. Translating regimens from models to humans faces scaling challenges.

## 4 Translational barriers

### 4.1 Individual response heterogeneity

Fasting responses vary dramatically across individuals. Genetic background plays an important role, with polymorphisms in nutrient-sensing pathways, circadian genes, and metabolic regulators modulating responses. Studies in genetically diverse mouse populations reveal that dietary restriction effects on cognition, metabolism, and lifespan vary widely (64). Sex differences represent a particularly important source of heterogeneity; females show distinct metabolic and hormonal responses (66, 67). The historical exclusion of females from both preclinical and clinical studies represents a significant gap.

Age influences fasting responses, with effects differing when interventions are initiated at different ages (37, 72). Older adults may be more susceptible to lean mass loss and electrolyte disturbances. Baseline metabolic status substantially modulates responses; individuals with obesity or insulin resistance often show greater improvements but also higher variability. It is critical to note that many metabolic improvements may be partially attributable to weight loss and reduced caloric intake rather than fasting-specific timing effects. Gut microbiome composition predicts metabolic responses (73). Together, these sources suggest universal fasting recommendations are unlikely optimal and personalized approaches are needed.

### 4.2 Long-term safety and adaptation

Long-term safety profile remains inadequately characterized. Lean mass depletion is a particular concern, especially in older adults, because prolonged fasting may accelerate sarcopenia (74). Electrolyte disturbances including hyponatremia and hypokalemia can occur during extended fasting (75). Endocrine effects have received limited attention; menstrual cycle disruption has been reported. Compensatory adaptations including hyperphagia during refeeding and reduced resting energy expenditure may undermine sustained benefits (76). Psychological effects require systematic investigation. The absence of prospective long-term studies with systematic adverse event monitoring represents a fundamental evidence gap.

### 4.3 Methodological and biomarker limitations

Mechanistic research in humans is severely constrained by inability to non-invasively quantify core fasting responses. Inability to quantify *in vivo* autophagic flux non-invasively is particularly significant (77). Surrogate markers provide indirect mechanistic inference with limited specificity. Tissue inaccessibility represents another fundamental limitation; critical mechanistic data from brain, liver, and immune compartments are rarely obtainable. Development of non-invasive techniques would substantially enhance mechanistic understanding (78, 79).

### 4.4 Clinical readiness assessment

*Sufficiently established for clinical consideration*: Weight loss and metabolic improvement (insulin sensitivity, glycemic control, lipid profile) in overweight/obese individuals with short-term interventions (≤6 months). Time-restricted eating appears feasible with acceptable adherence.

*Promising but requires further evidence*: Long-term weight maintenance, cardiovascular event reduction, diabetes prevention or remission, neuroprotection, and cancer-related benefits. Definitive trials with hard clinical endpoints are lacking.

*Remains uncertain*: Optimal fasting protocol for specific populations and outcomes; individual response determinants; long-term safety in diverse populations; mechanisms mediating benefits in humans; comparative effectiveness versus other interventions.

*Not recommended*: Extended fasting (>48 h) without medical supervision; fasting during pregnancy, lactation, or in individuals with eating disorders, malnutrition, or advanced frailty; use as substitute for evidence-based medical treatments; general population recommendations without consideration of individual factors.

## 5 A prioritized research agenda

### 5.1 Priority 1: Establish mechanistic hierarchy and causality

Inducible tissue-specific knockout models for AMPK, SIRT1, mTORC1, and autophagy genes should determine which pathways are required for specific benefits. Temporal profiling using multi-omics across fasting and refeeding phases can identify sequential activation patterns. Comparative studies of IF, PF, and pharmacological pathway activators can distinguish pathway-specific contributions. (80).

### 5.2 Priority 2: Address translational Fidelity

Large-scale human multi-omic studies with deep phenotyping during standardized fasting protocols are needed to characterize molecular responses and validate mechanisms identified in preclinical models (81). Tissue-biopsy studies in muscle and adipose tissue can directly validate mechanisms (82). Machine learning approaches integrating multi-omic data with clinical outcomes can identify robust biomarkers. Standardized protocols for human fasting studies are essential for enabling cross-study comparison.

### 5.3 Priority 3: Resolve individual response determinants

Genome-wide association studies in large fasting cohorts can identify genetic variants associated with differential responses. Microbiome profiling with metagenomic sequencing can identify baseline microbial features predicting responses. Machine learning approaches integrating genetic, metabolic, microbiome, and clinical data can develop predictive models. Genotype-directed intervention studies can test whether personalized protocols improve outcomes.

### 5.4 Priority 4: Establish long-term safety and efficacy

Prospective longitudinal cohorts with systematic adverse event monitoring are needed to characterize long-term effects. Trials with hard clinical endpoints including cardiovascular events, diabetes incidence, cancer incidence, and mortality are necessary. Subgroup analyses in vulnerable populations are essential. International fasting registries would accelerate accumulation of long-term safety data.

### 5.5 Priority 5: Develop and validate biomarkers

Biomarker development should prioritize markers reflecting mechanistic engagement of key pathways including autophagy flux, mitochondrial function, NAD^+^ metabolism, and immune cell function. Validation of circulating markers of autophagy flux against tissue-based measures would enable non-invasive monitoring. Development of PET tracers for *in vivo* imaging would provide unprecedented insight. Standardized protocols for biomarker measurement across studies would enable cross-study comparison.

## 6 Methodological recommendations

### 6.1 Recommendations for preclinical studies

Preclinical studies should employ aged, diseased, and genetically diverse models to enhance translational relevance. The use of aged rodents is particularly important for understanding how fasting affects the biology of aging, as responses may differ substantially between young and old animals. Disease models, including APP/PS1 mice for Alzheimer’s disease, diet-induced obesity and NAFLD models, and cancer models, can reveal disease-specific mechanisms and therapeutic effects. Genetically diverse strains, such as the Diversity Outbred or Collaborative Cross populations, can capture the range of genetic variation present in human populations and identify genetic determinants of fasting responses (64).

Both sexes should be included in preclinical studies, and sex-specific analyses should be reported to characterize sex differences in fasting responses. The standardization of fasting protocols, including duration, frequency, timing, and refeeding composition, would enable cross-study comparison and meta-analysis across laboratories. Studies that explicitly distinguish the effects of timing versus caloric intake are essential for understanding the independent contributions of fasting duration and energy reduction.

The development of reporting guidelines for preclinical fasting studies, including recommended metrics for fasting compliance and metabolic monitoring, would enhance the quality and reproducibility of preclinical research. The use of appropriate controls, including ad libitum fed groups and pair-fed controls, is essential for distinguishing fasting-specific effects from those related to reduced caloric intake.

### 6.2 Recommendations for human studies

Human studies should conduct longer-duration trials with adequate power to detect clinically meaningful effects. While short-term studies provide valuable mechanistic insights, long-term trials are essential for establishing efficacy and safety. Studies of 6 months or longer are needed to assess sustained effects and to capture the full trajectory of adaptation and response.

Diverse populations should be included across age, sex, ethnicity, and comorbidity status to ensure generalizability of findings. Controlled feeding designs, in which all food is provided to participants, should be employed where feasible to control for the confounding effects of dietary composition and total caloric intake (83). Studies that explicitly compare fasting protocols to continuous caloric restriction matched for total energy deficit are essential for isolating timing-specific effects from those driven by weight loss. Without such comparisons, it remains unclear whether fasting provides unique benefits beyond those achievable through conventional dietary restriction.

Standardized outcome measures should be used across trials to enable comparison and meta-analysis, with core outcome sets developed through international consensus. The inclusion of appropriate control groups, such as continuous caloric restriction or usual diet, is essential for distinguishing the specific effects of fasting from general weight loss or health behavior change. Long-term follow-up after intervention completion can assess the durability of benefits and identify potential rebound effects.

### 6.3 Recommendations for reporting standards

The quality and comparability of fasting research would be substantially enhanced by improved reporting standards. Clear descriptions of fasting protocols, including duration, frequency, timing, refeeding composition, and compliance assessment, should be required for all studies. Adherence rates and compliance metrics should be reported in a standardized manner to assess the feasibility and real-world applicability of fasting interventions.

The distinction between primary and secondary outcomes should be clearly specified, with pre-registration of primary analyses to reduce the risk of selective reporting. Sex-disaggregated and subgroup analyses should be reported where sample sizes permit, enabling the identification of differential effects across population subgroups. The reporting of adverse events, including both serious and non-serious events, should be systematic and comprehensive to characterize safety profiles.

The development of a CONSORT extension for fasting trials, similar to extensions for other interventions, would provide structured guidance for reporting and would enhance the quality and transparency of clinical trial reports. Pre-registration of studies, including detailed protocols and analysis plans, should be standard practice to reduce bias and improve transparency. The sharing of individual participant data through open science repositories would enable secondary analyses and meta-analyses that can identify effects not detectable in individual studies.

## 7 Conclusion

IF and PF represent biologically plausible interventions for healthspan extension, supported by substantial evidence of pathway activation and multi-system benefits across preclinical models and human studies. However, the current literature is characterized by excessive breadth without sufficient depth, descriptive cataloging without critical evaluation, and positive emphasis without adequate attention to uncertainty and limitations. The field has successfully established biological plausibility and generated extensive descriptive data, but it has not yet achieved the conceptual and methodological maturity required for confident clinical translation.

This review proposes that advancing the field requires moving beyond enumeration of molecular pathways toward a hierarchical mechanistic framework that distinguishes primary drivers from secondary consequences and identifies areas of genuine mechanistic uncertainty. The metabolic switch from glucose to ketone metabolism, AMPK activation, and mTOR suppression represent well-established primary responses that are consistently observed across species and are supported by high-quality mechanistic evidence. Autophagy induction, mitochondrial remodeling, Nrf2 activation, and inflammation resolution are secondary adaptations with strong preclinical support but limited human validation, representing probable mechanisms that require further investigation to confirm their role in human physiology. Numerous speculative mechanisms, including epigenetic memory, xenohormesis, and microbiome causality, require substantially more evidence before they can be confidently incorporated into mechanistic models or targeted for therapeutic intervention. The distinction between well-established, probable, and speculative mechanisms is essential for maintaining scientific rigor and avoiding overinterpretation of preliminary findings.

The central translational barriers are not primarily mechanistic knowledge gaps but rather the limited generalizability of animal models, the methodological heterogeneity of human trials that complicates cross-study comparison, insufficient long-term safety data in diverse populations, and the absence of validated biomarkers to monitor mechanistic engagement and predict individual responses. Addressing these barriers requires a research agenda focused on mechanistic causality, translational fidelity, individual heterogeneity, long-term outcomes, and biomarker development. This agenda should prioritize studies that establish causation rather than correlation, that validate mechanisms in human tissues rather than assuming conservation from model systems, and that characterize the full range of individual variability rather than averaging across diverse populations.

Having critically evaluated the available evidence, we offer our own stratified judgment on clinical readiness. Time-restricted eating (TRE) as a feasible dietary strategy for modest weight loss and metabolic improvement (insulin sensitivity, glycaemic control, lipid profile) in overweight or obese adults over ≤6 months is sufficiently supported for clinical consideration, based on consistent RCT evidence showing acceptable adherence and generally mild adverse events. Conversely, fasting-mimicking diets are promising but experimental for applications including metabolic health and chemoprotection, neuroprotection (e.g., BDNF elevation), and long-term prevention of cardiovascular events or diabetes, all of which require definitive trials with hard clinical endpoints. Finally, practices that warrant caution or are not recommended include extended fasting (>48 h) without medical supervision; use during pregnancy, lactation, eating disorders, malnutrition, or advanced frailty; substitution for evidence-based medical treatments; and universal recommendations without consideration of individual genetic, metabolic and behavioral factors.

In conclusion, we emphasize that the field’s next phase must prioritize rigorous hypothesis testing, methodological standardization, mechanistic causality, and human validation over model extrapolation. Enthusiasm must be tempered by scientific objectivity, and the substantial individual variability in responses must be acknowledged in both clinical guidelines and public health messaging.

## Glossary

AMPK: Adenosine monophosphate-activated protein kinase
ARE: Antioxidant-response element
APP/PS1: Amyloid precursor protein/Presenilin-1
ATG5: Autophagy-related gene 5
ATP: Adenosine triphosphate
BDNF: Brain-derived neurotrophic factor
BHB: β-hydroxybutyrate
***C. elegans***: *Caenorhabditis elegans*
CONSORT: Consolidated Standards of Reporting Trials
CR: Caloric restriction
FGF21: Fibroblast growth factor 21
FMD: Fasting-mimicking diet
FMT: Fecal microbiota transplantation
FOXO: Forkhead box O
GH: Growth hormone
HDAC: Histone deacetylase
IGF-1: Insulin-like growth factor 1
IF: Intermittent fasting
IL-1β: Interleukin-1 beta
IL-6: Interleukin-6
IL-10: Interleukin-10
LAMP2: Lysosomal-associated membrane protein 2
LC3B: Microtubule-associated protein 1 light chain 3-beta
mTOR: Mechanistic target of rapamycin
mTORC1: mTOR complex 1
NAD^+^: Nicotinamide adenine dinucleotide
NADH: Reduced form of nicotinamide adenine dinucleotide
NAFLD: Non-alcoholic fatty liver disease
NF-κB: Nuclear factor kappa B
NLRP3: NLR family pyrin domain containing 3
Nrf2: Nuclear factor erythroid 2-related factor 2
p53: Tumor protein p53
p62: Sequestosome-1
PBMCs: Peripheral blood mononuclear cells
PET: Positron-emission tomography
PGC-1α: Peroxisome proliferator-activated receptor gamma coactivator 1-alpha
PKA: Protein kinase A
PRKAA1: Protein kinase AMP-activated catalytic subunit alpha 1
PF: Periodic fasting
RCT: Randomized controlled trial
RHEB-1: Ras homolog enriched in brain-1
ROS: Reactive oxygen species
SCFA: Short-chain fatty acid
SIRT1: Sirtuin 1
SIRT3: Sirtuin 3
SOD: Superoxide dismutase
TNF-α: Tumor necrosis factor-alpha
TRE: Time-restricted eating
